# Clinical characteristics of patients with bipolar disorder and premorbid traumatic brain injury: a cross-sectional study

**DOI:** 10.1186/s40345-018-0128-6

**Published:** 2018-09-10

**Authors:** Ole Kristian Drange, Arne Einar Vaaler, Gunnar Morken, Ole Andreas Andreassen, Ulrik Fredrik Malt, Per Ivar Finseth

**Affiliations:** 1 Department of Mental Health, Norwegian University of Science and Technology, Trondheim, Norway; 2 Department of Østmarka, Division of Mental Health Care, St. Olavs Hospital, Trondheim University Hospital, Trondheim, Norway; 3 Institute of Clinical Medicine, University of Oslo, Oslo, Norway; 4 NORMENT, Division of Mental Health and Addiction, Oslo University Hospital, Oslo, Norway; 5 Department of Research and Education, Division of Clinical Neuroscience, Oslo University Hospital, Oslo, Norway; 6 Department of Brøset, Division of Mental Health Care, St. Olavs Hospital, Trondheim University Hospital, Trondheim, Norway

**Keywords:** Bipolar disorder, Brain injuries, Diagnosis, Principal component analysis, Irritable mood, Migraine disorders

## Abstract

**Background:**

About one in ten diagnosed with bipolar disorder (BD) has experienced a premorbid traumatic brain injury (TBI), while not fulfilling the criteria of bipolar and related disorder due to another medical condition (BD due to TBI). We investigated whether these patients have similar clinical characteristics as previously described in BD due to TBI (i.e. more aggression and irritability and an increased hypomania/mania:depression ratio) and other distinct clinical characteristics.

**Methods:**

Five hundred five patients diagnosed with BD type I, type II, or not otherwise specified, or cyclothymia were interviewed about family, medical, and psychiatric history, and assessed with the Young Mania Rating Scale (YMRS) and the Inventory of Depressive Symptoms Clinician Rated 30 (IDS-C_30_). Principal component analyses of YMRS and IDS-C_30_ were conducted. Bivariate analyses and logistic regression analyses were used to compare clinical characteristics between patients with (n = 37) and without (n = 468) premorbid TBI.

**Results:**

Premorbid TBI was associated with a higher YMRS disruptive component score (OR 1.7, 95% CI 1.1–2.4, p = 0.0077) and more comorbid migraine (OR 4.6, 95% CI 1.9–11, p = 0.00090) independently of several possible confounders. Items on disruptive/aggressive behaviour and irritability had the highest loadings on the YMRS disruptive component. Premorbid TBI was not associated with an increased hypomania/mania:depression ratio.

**Conclusions:**

Disruptive symptoms and comorbid migraine characterize BD with premorbid TBI. Further studies should examine whether the partial phenomenological overlap with BD due to TBI could be explained by a continuum of pathophysiological effects of TBI across the diagnostic dichotomy.

*Trial registration* ClinicalTrials.gov: NCT00201526. Registered September 2005 (retrospectively registered)

**Electronic supplementary material:**

The online version of this article (10.1186/s40345-018-0128-6) contains supplementary material, which is available to authorized users.

## Background

About one in ten diagnosed with bipolar disorder (BD) has experienced a premorbid traumatic brain injury (TBI) (Sagduyu 2002; Orlovska et al. 2014; Chi et al. 2016). An association between TBI and risk of BD is established by nation-wide cohort studies from Denmark (Orlovska et al. 2014) and Taiwan (Chi et al. 2016) and a meta-analysis of prior smaller studies (Perry et al. 2016). The nature of this association could theoretically include inverse causation, confounding, and causation (Malaspina et al. 2001). A review on the topic using Bradford Hill’s criteria finds evidence for a causal relationship in some cases (van Reekum and Cohen 2000). The American Psychiatric Association also acknowledges that TBI can cause BD (American Psychiatric Association 2013).

The Diagnostic and Statistical Manual of Mental Disorders 5th edition (DSM 5) distinguishes between a diagnosis of BD and a diagnosis of bipolar and related disorder due to another medical condition (in the context of this paper abbreviated to BD due to TBI) (American Psychiatric Association 2013). To fulfil the criteria of the latter, there must be evidence from the history (e.g. an acute or subacute onset of the mood disturbance after the onset of the general medical condition), physical examination, or laboratory findings that the mood disturbance is the direct physiological consequence of the general medical condition. Similar criteria exist for the corresponding diagnosis in the DSM fourth edition (DSM IV) (American Psychiatric Organization 1994). Thus, among patients with symptoms of BD, a premorbid TBI does not per se qualify for a diagnosis of BD due to TBI.

The validity of this dichotomy between BD *with* premorbid TBI and BD *due to* TBI is questionable (Kendell and Jablensky 2003). First, BD has a non-absolute monozygotic concordance (Craddock and Sklar 2013). Environmental factors must thus be of some etiological importance in both diagnoses. Second, a latency in the onset of BD after TBI does not exclude a pathophysiological impact of the latter. In a nation-wide sibling-controlled cohort study from Sweden (Sariaslan et al. 2016), TBI predicted risks of later psychiatric visits and hospitalizations. The risks were also increased among those with TBI in childhood and of mild severity. Even though these findings were not stratified by diagnoses, they highlight the possibility of a causative effect of TBI in childhood on psychiatric disorders in adulthood. Third, TBI can induce a range of pathophysiological processes which are not necessarily detected by standard radiological or electrophysiological assessment (Mayer et al. 2010; Nuwer et al. 2005; Blennow et al. 2012).

It is thus possible that TBI could have had an impact in the pathophysiological trajectory of BD in some patients who do not fulfil the criteria of BD due to TBI. If so, these patients would be expected to have some of the same clinical characteristics as previously described in BD due to TBI. Such clinical characteristics are more aggression and irritability, and a high hypomania/mania:depression ratio (Shukla et al. 1987; Jorge et al. 1993). Clinical characteristics of BD with premorbid TBI could obviously be influenced by other factors than the TBI. Family history of mental illness is particularly of relevance, since it is associated with both risk of TBI (Malaspina et al. 2001) and more severe clinical characteristics of BD (Antypa and Serretti 2014). Also, some clinical characteristics (e.g. levels of aggression and irritability) could be sensitive to potential differences between those with and without premorbid TBI in regard to BD subtypes (Baek et al. 2011; Serretti and Olgiati 2005), and mood states (Young et al. 1978) and pharmacological treatment (Müller-Oerlinghausen and Lewitzka 2010) at assessment.

The aim of the present study was to identify clinical characteristics in patients with BD and premorbid TBI, using a large sample of patients from psychiatric care. We hypothesized to find more aggression and irritability, a higher hypomania/mania:depression ratio, and other clinical characteristics distinguishing patients with premorbid TBI from those without. Further, we hypothesized that eventual findings would be independent of possible confounders.

## Methods

### Study design

The study has a cross-sectional design.

### Setting

Psychiatric health care services in Norway are publicly funded and the hospitals have catchment area responsibilities. From 2003 to 2012 data were collected from 17 psychiatric hospitals and outpatient clinics collaborating in the Bipolar Research And Innovation Network Norway (BRAIN) study (Morken et al. 2009).

### Participants

Patients diagnosed with BD type I, type II, or not otherwise specified (NOS), or cyclothymia were invited to participate. Diagnoses were confirmed by trained clinicians with the Structured Clinical Interview for DSM IV Axis I Disorders (SCID I) (First et al. 1997) or the Mini-International Neuropsychiatric Interview (M.I.N.I.) (Sheehan et al. 1998). The only exclusion criterion was inability to give informed consent. There is no information available on the number of patients who refused to participate. A total of 549 patients were included. The present study consists of the 505 patients who answered the questions about TBI and age at onset of BD.

### Data sources

All patients were assessed with a Norwegian adaptation of the Network Entry Questionnaire (NEQ). NEQ is a semi-structured interview developed by the Bipolar Collaboration Network (Leverich et al. 2001). The Norwegian adaptation of NEQ was used to obtain data on TBI, demographic factors, family history, course of illness, pharmacological treatment, somatic comorbidity, and psychiatric comorbidity in childhood. The Young Mania Rating Scale (YMRS) (Young et al. 1978) and the Inventory of Depressive Symptomatology Clinician Rated 30 (IDS-C_30_) (Rush et al. 1996) were used to obtain data on symptom composition and burden. The SCID I or the M.I.N.I. were used to obtain data on current mood state.

### Variables

The grouping variable was premorbid TBI. Patients were asked if they had had a head injury with loss of consciousness (LOC) and if they had had a head injury without LOC. The questions could be answered *yes (diagnosed)*, *no*, or *don’t know*. An answer of *yes (diagnosed)* thus implicated a prior diagnostic evaluation of a general practitioner or hospital doctor. In Norway, head injuries are in general practice defined according to the International Classification of Primary Care (ICPC) (Brage et al. 1996; Lamberts and Wood 1987), and in hospital settings according to the International Classification of Diseases (ICD) 10th revision (World Health Organization 1993). An ICPC diagnosis of head injury (N79–N80) requires loss of consciousness, neurological sequela, or cerebral injury, while an ICD-10 diagnosis of head injury implies that the criteria for hospital admission (i.e. loss of consciousness, amnesia, reduced responsiveness, focal neurological deficits, or Glasgow Coma Scale Score < 15) in Norway have been met (Lamberts and Wood 1987; Ingebrigtsen et al. 2000). If answering *yes (diagnosed)*, patients where further asked about their age at the time of diagnosis. We defined premorbid TBI as having received a diagnosis of head injury of any severity at an age lower than age at onset of BD. The definition of age at onset of BD was age at first depressive symptoms associated with dysfunction or first hypomanic or manic symptoms similar to those experienced in adulthood (Morken et al. 2009).

Years of education refers to the accumulated time spent at upper secondary schools, university colleges, and universities.

From variables of family history of mental illness, we computed answers of *yes (diagnosed)* as positive and *probable* or *no* as negative to questions of schizophrenia, BD, and depression in 1st degree relatives.

Duration of illness was calculated as the difference between age at inclusion and age at onset. Variables on affective episodes per year were calculated as fractions of number of episodes above duration of illness. Rapid cycling was dichotomized from a question of four or more episodes per year during lifetime, where answers of *yes* were defined as positive and *no* or *uncertain* were defined as negative. The variable on serious suicidal attempts was defined by lifetime history of suicidal attempts leading to medical assistance, emergency room visit, or hospitalization.

Variables on pharmacological treatment were defined by current use of lithium, anticonvulsants, antipsychotics, and antidepressants.

History of epileptic seizures and comorbid migraine were defined by answers of *yes (diagnosed)* to questions of lifetime history of the two, while answers of *no* or *don’t know* were regarded as negative. Obesity was defined as body mass index ≥ 30 kg/m^2^ as calculated from self-reported height and weight. Psychiatric comorbidity was defined by one variable on abuse or dependence of alcohol and a similar variable on other substances, both defined by SCID-I or M.I.N.I., as well as one variable on self-reported diagnosis of childhood attention deficit (hyperactivity) disorder (AD(H)D) obtained from the NEQ.

Variables on symptom composition and burden were obtained from principal components and total scores, respectively, of YMRS and IDS-C_30_ at inclusion (see statistical methods).

### Bias

As an indirect test of bias in self-reported variables, we compared the groups on a four-point ordinal item in the NEQ on the clinicians’ judgement of the reliability of the patients’ answers. The item scores ranged from 1 to 4, corresponding to very reliable and unreliable answers, respectively.

### Statistical methods

R (version 3.4.0) (2018) and RStudio (version 1.0.143) (2018) were used for statistical analyses.

The R package psych was used for principal component analyses (PCA) of items in the YMRS and IDS-C_30_. The purpose of conducting a PCA was to limit the number of comparisons in analyses of symptomatology. Items on appetite and weight in the IDS-C_30_ are pair-wise optional and were excluded to avoid large amounts of missing data. Cronbach’s alpha was used to test the internal reliability of the rating scales. A Kaiser-Meyer-Olkin (KMO) test was used to measure sample size adequacy. A determinant cut-off at > 0.00001 was used to avoid multicollinearity. The Velicer’s map criterion was used to determine the number of components to retain. Direct oblimin oblique rotation was used since components were assumed to be correlated. Pattern matrices of component loadings > 0.4 were used to interpret the results. Standardized component scores were saved for each participant.

Bivariate analyses of differences between the groups were undertaken for all variables. Pearson’s Chi squared tests, Fisher’s exact tests, Mann–Whitney U-tests, and independent samples t-tests were used as appropriate. The alpha level was set to < 0.05. A Bonferroni correction of 40 comparisons, two of which were not included in the paper due to large amounts of missing data, yielded a p-value threshold of < 0.0013. The beta level for each comparison was not calculated.

A backward likelihood ratio logistic regression analysis was conducted in order to find clinical characteristics associated with premorbid TBI independent of each other and possible confounders. We included variables with p-values < 0.1 from bivariate analyses, and possible confounding variables based on the literature and clinical experience. These were age, sex, years of education, 1st degree family members with schizophrenia, BD, or depression, rapid cycling, migraine, AD(H)D in childhood, comorbid alcohol abuse or dependence, comorbid substance abuse or dependence, YMRS total score, IDS-C_30_ total score, YMRS disruptive component score, and IDS-C_30_ activated component score. Depressive episodes per year and hypomanic episodes per year were not included due to large amounts of missing data. YMRS total score had a skewed distribution with large amounts of null scores. Logarithmic transformation of total score plus one was therefore used in the regression model.

Further, we built additional logistic regression models to test whether our findings from the backward likelihood ratio logistic regression analysis were dependent on BD subtypes, mood states, and pharmacological treatments. First, we built a base model (Model 1) including sociodemographic factors (age, gender, and years of education), family history of mental illness (schizophrenia, BD, and depression), and clinical characteristics significantly associated with premorbid TBI in the backward likelihood ratio logistic regression analysis (YMRS disruptive component score and comorbid migraine) as independent variables. Then, we added and removed dummy variables of BD subtypes (Model 2a: type 1 vs. others; Model 2b: type 2 vs. others), mood states (Model 3a: depressed vs. others; Model 3b: euthymic vs. others etc.), and pharmacological treatments (Model 4a: lithium vs. others; Model 4b: anticonvulsants vs. others etc.) in successive models.

Variance inflation factors (VIF) between all independent variables were calculated to assess for multicollinearity.

## Results

### Sample characteristics

Five hundred five patients were included. Mean age was 42 years (SD 14), 286 (57%) were women, and 256 (53%) had BD type I. Thirty-seven patients (7.3%) reported a premorbid TBI, of whom 23 (4.6%) had LOC. Age at TBI and years from age at TBI to age at onset of BD had mean values of 10 years (SD 9.0) and 8.9 years (SD 7.3), respectively.

Clinicians judged the reliability of the patients’ answers as high in both groups (median 1 vs. 1 (interquartile range 1 vs. 1), p = 0.45).

### Principal component analyses

Cronbach’s alpha was 0.90 for both YMRS and IDS-C_30_, which implicates a very good internal reliability of the rating scales.

Two components were extracted from items in both YMRS (Table 1) and IDS-C_30_ (Table 2). The KMO measure of sampling adequacy was 0.89 and 0.93, respectively. Barlett’s test of spherity was significant for both. The determinant was 0.0017 and 0.000029, respectively. The components accounted for 62% of the variance in the YMRS and 39% of the variance in the IDS-C_30_.

**Table 1:** Items loadings on principal components of the Young Mania Rating Scale

Item	Components (variance explained)	
	Elated (37%)	Disruptive (24%)
Elevated mood	0.95	
Increased motor activity/energy	0.93	
Speech	0.84	
Language/thought disorder	0.75	
Sexual interest	0.70	
Content	0.56	
Disruptive/aggressive behavior		0.89
Irritability		0.76
Appearance		0.69
Insight		0.65

**Table 2:** Item loadings on principal components of the Inventory of Depressive Symptomatology Clinican Rated 30

Item	Components (variance explained)	
	Depressed (31%)	Activated (9%)
Energy/fatiguability	0.86	
Pleasure/enjoyment	0.86	
Involvement	0.85	
Mood (sad)	0.78	
Outlook (future)	0.78	
Reactivity of mood	0.74	
Psychomotor slowing	0.68	
Sexual interest	0.67	
Outlook (self)	0.64	
Suicidal ideation	0.64	
Mood (anxious)	0.64	
Leaden paralysis/physical energy	0.63	
Concentration/decision making	0.53	
Mood variation	0.41	
Psychomotor agitation		0.60
Mid-nocturnal insomnia		0.60
Early morning insomnia		0.60
Sleep onset insomnia		0.53
Mood (irritable)		0.41

### Bivariate analyses

By applying bivariate analyses with correction for multiple testing, we found more comorbid migraine (36 vs. 13%, p = 0.00030) among patients with premorbid TBI (Table 3).

**Table 3:** Demographics, family history, and clinical characteristics in patients with and without premorbid traumatic brain injury

	All (n = 505)	+pTBI (n = 37)	−pTBI (n = 468)	p-value
Demographics				
Age	505	41 (15)	42 (13)	0.74^a^
Female sex	505	21/37 (57)	265/468 (57)	1.0^c^
Years of education	495	4.8 (3.1)	4.4 (3.1)	0.55^b^
Family history				
Schizophrenia	496	4/37 (11)	9/459 (2.0)	0.012^d^
Bipolar disorder	497	5/37 (14)	105/460 (23)	0.27^c^
Depression	497	26/37 (70)	199/460 (43)	0.0027^c^
Any	497	27/37 (73)	247/460 (54)	0.036^c^
Mood state				
Depressed	504	28/37 (76)	305/467 (65)	0.27^c^
Euthymic	504	1/37 (2.7)	12/467 (2.6)	1.0^c^
Mixed	504	1/37 (2.7)	22/467 (4.7)	0.88^c^
Hypomanic	504	0/37 (0)	10/467 (2.1)	0.77^c^
Manic	504	7/37 (19)	118/467 (25)	0.51^c^
Course of illness				
Age at onset	505	20 (12)	19 (11)	0.81^b^
Duration of illness	505	21 (14)	23 (14)	0.45^b^
Bipolar disorder subtype	505			0.57^d^
Type I		18/37 (49)	247/468 (53)	
Type II		18/37 (49)	213/468 (46)	
Cyclothymia		0/37 (0)	1/468 (2)	
Not otherwise specified		1/37 (3)	7/468 (1)	
Depressive episodes per year	420	1.3 (1.8)	0.78 (1.1)	0.014^b^
Hypomanic episodes per year	424	1.4 (2.0)	0.92 (2.2)	0.025^b^
Manic episodes per year	461	0.073 (0.15)	0.091 (0.21)	0.23^b^
Hypomania/mania:depression ratio	405	1.3 (1.1)	2.1 (5.2)	0.59^b^
Admissions per decade	449	0.82 (1.1)	1.4 (2.6)	0.24^b^
Rapid cycling	491	14/35 (40)	103/456 (23)	0.034^c^
Serious suicidal attempt(s)	500	14/37 (38)	202/463 (44)	0.61^c^
Current pharmacological treatment				
Lithium	483	12/33 (36)	102/450 (23)	0.11^c^
Anticonvulsants	483	20/33 (61)	302/450 (67)	0.57^c^
Antipsychotics	483	19/33 (58)	270/450 (60)	0.93^c^
Antidepressants	483	12/33 (36)	165/450 (37)	1.0^c^
Comorbidity				
Epileptic seizures	505	1/37 (2.7)	9/468 (1.9)	0.54^d^
Migraine	501	13/36 (36)	59/465 (13)	0.00030^c^*
Obesity	498	4/36 (11)	79/462 (17)	0.49^c^
Alcohol, abuse or dependence	505	5/37 (14)	64/468 (14)	1.0^c^
Other substances, abuse or dependence	505	2/37 (5.4)	28/468 (6.0)	1.0^d^
Childhood AD(H)D	497	6/37 (16)	33/460 (7.2)	0.059^c^
Symptom composition				
YMRS elated component score	484	0.12 (1.2)	− 0.020 (0.98)	0.77^b^
YMRS disruptive component score	484	0.54 (1.7)	− 0.050 (0.90)	0.0044^b^
IDS-C_30_ depressed component score	446	0.19 (1.0)	0.011 (1.0)	0.30^b^
IDS-C_30_ activated component score	446	0.21 (0.86)	− 0.046 (0.99)	0.041^b^
Symptom burden				
YMRS total score	497	7.9 (11)	5.4 (7.8)	0.16^b^
IDS-C_30_ total score	490	28 (14)	24 (15)	0.087^b^

Results are presented by fraction (%) for categorical variables and mean (SD) for continuous variables. +pTBI premorbid traumatic brain injury. −pTBI no premorbid traumatic brain injury

* Multiple comparisons p-value threshold = 0.0013

^a^Independent samples t-test

^b^Mann–Whitney-U test

^c^Pearson’s Chi squared test

^d^Fisher’s exact test

### Logistic regression analyses

Data for 395 of 505 participants (78%) were complete and thereby included in the backward likelihood ratio logistic regression analysis. Among clinical characteristics, the YMRS disruptive component score (OR 1.7, 95% CI 1.1–2.4, p = 0.0077) and comorbid migraine (OR 4.6, 95% CI 1.9–11, p = 0.00090) were independently associated with premorbid TBI (Table 4). Family history of schizophrenia (OR 11, 95% CI 2.3–51, p = 0.0025) was also independently associated with premorbid TBI.

**Table 4:** Final step of the backward likelihood ratio logistic regression analysis on variables independently associated with premorbid traumatic brain injury among patients with bipolar disorder

	Coefficient	S.E.	OR	95% CI	Wald Z	p-value
Intercept	− 1.2	0.36	0.29	0.14–0.60	− 3.4	0.00078
Family history schizophrenia	2.4	0.79	11	2.3–51	3.0	0.0025
Comorbid migraine	1.5	0.46	4.6	1.9–11	3.3	0.00090
YMRS disruptive component	0.51	0.19	1.7	1.1–2.4	2.7	0.0077

*S.E.* standard error, *OR* odds ratio, *CI* confidence interval

The associations between premorbid TBI and YMRS disruptive component score and comorbid migraine remained significant after correction for BD subtypes, mood states, and pharmacological treatments (Additional file 1: Table S1).

Nagelkerke R^2^ of 0.31 implied a low relationship between prediction and grouping in the backward likelihood ratio logistic regression analysis. VIF was < 3.0 between all independent variables in all logistic regression models.

## Discussion

We found that premorbid TBI was associated with comorbid migraine in bivariate analyses with correction for multiple comparisons. In a logistic regression model, we found that premorbid TBI was associated with disruptive symptoms and comorbid migraine independently of a range of possible confounders. Family history of schizophrenia was also independently associated with premorbid TBI. We did not find associations between premorbid TBI and any other clinical characteristics and specifically not with hypomania/mania:depression ratio.

The finding of more disruptive symptoms corresponds with our hypothesis of finding similar clinical characteristics in BD with premorbid TBI as previously described in BD due to TBI (Shukla et al. 1987; Jorge et al. 1993). Aggression and irritability, which in our study had the highest loadings on the disruptive component, are common symptoms after TBI of all severities (Lique Sté Fan and Mathé 2015; Hammond et al. 2016). A cross-sectional study on 89 patients with and without aggression after TBI of all severities, found that aggression was associated with premorbid substance use disorders, affective disorders, and poor social functioning (Tateno et al. 2003). Similarly, a study on 97 children with severe TBI found that preinjury affective lability and psychosocial adversity predicted affective lability (including irritability and temper outbursts) at one year follow-up (Vasa et al. 2015). These studies suggest that TBI could result in more disruptive symptoms among patients with a disposition to affective symptoms and lower social function/support. Further, aggression after TBI was in the same studies associated with frontal lobe lesions (Tateno et al. 2003; Vasa et al. 2015), highlighting a possible structural substrate for disruptive symptoms after TBI. Frontal lobe regions are also implicated in mania secondary to focal brain lesions in general (Satzer and Bond 2016), and are on average thinner among patients with BD than among healthy controls (Hibar et al. 2018).

The mean YMRS score in the premorbid TBI group in our study was low. Further, the association between the YMRS disruptive component score and premorbid TBI remained significant after corrections for BD subtypes, mood states, and pharmacological treatments (Additional file 1: Table S1). It is thus a possibility that the disruptive symptoms in most patients represent a personality trait related to premorbid TBI rather than a mood state related to BD. However; disruptive symptoms after TBI could also be rapidly alternating (Arciniegas and Wortzel 2014) and thereby resemble, and possibly fulfil the criteria of, short lasting manic states (Coetzer 2008).

Our results are in line with previous findings that migraine is a common headache after TBI (Lucas et al. 2014; Stacey et al. 2017). Inflammation and impaired pain modulation after TBI are two of the suggested pathophysiological mechanisms (Ruff et al. 2016). Furthermore, migraine and BD are bi-directionally associated (Fornaro et al. 2015) and possibly influenced by shared environmental and genetic factors (Sucksdorff et al. 2016). It is thus difficult to conclude on the nature behind our finding of an association between migraine and premorbid TBI among patients with BD.

Although it was not a primary outcome of our study, our finding of more family history of schizophrenia among those with premorbid TBI harmonizes with findings in a study of Malaspina et al. (Malaspina et al. 2001). They assessed for TBI in members of families where at least two 1st degree-relatives had schizophrenia or BD. Members of the schizophrenia pedigrees had an increased risk of TBI which was independent of their own diagnoses. The effect of family history of schizophrenia on risk of premorbid TBI could be mediated by several factors. Examples are co-inherited motor coordination problems (Ismail et al. 1998; Burton et al. 2017), and co-inherited susceptibility to risk-taking behaviour (Strawbridge et al. 2018).

Regarding course of illness, the findings of more rapid cycling, depressive episodes per year and hypomanic episodes per year were not significant after correction for multiple testing. Due to missing data in the latter two variables, only rapid cycling was included in the regression model, and it was not independently associated with premorbid TBI. In the Systematic Treatment Enhancement Program for Bipolar Disorder (STEP-BD) study, mild TBI after age at onset of BD, but not before, was associated with rapid cycling (Sagduyu 2002). One of several possible explanations to our and the STEP-BD study’s findings is that the co-occurrence of TBI and more rapid cycling are epiphenomena of increased genetic susceptibility to BD (Antypa and Serretti 2014). We found a hypomania/mania:depression ratio of 1:1 in the premorbid TBI group, which was not higher than among those without premorbid TBI. In comparison, Shukla et al. reported a ratio of 15:1 in a study on patients with BD due to TBI (Shukla et al. 1987). Several differences between the samples, e.g. regarding clinical setting, severity of TBI, and frequency of comorbid epilepsy, make it difficult to conclude on the cause(s) of this discrepancy.

There were no differences between the groups on current treatment with lithium, anticonvulsants, antipsychotics or antidepressants. Previous studies have found increased treatment response to valproate among patients with BD and a history of TBI (Pope et al. 1988; Stoll et al. 1994). However, treatment response is not directly comparable to our measures, which could be influenced by the prescribing clinicians’ preferences and discontinuation due to side effects.

Epilepsy is previously suggested as the pathophysiological mechanism in BD due to TBI (van Reekum and Cohen 2000). In our study of BD with premorbid TBI, self-report did not unveil higher frequencies of epileptic seizures. Nevertheless, not all seizures are recognized by patients (Elger and Hoppe 2018).

The prevalence of premorbid TBI in our study from Norway (7.3%) was lower than in the STEP-BD study from the United States (10% with premorbid mild TBI) (Sagduyu 2002) and in register-based studies from Denmark (11%) (Orlovska et al. 2014) and Taiwan (25%) (Chi et al. 2016). However, both variation in prevalence of TBI across nations and different methodologies across studies (Corrigan et al. 2010) warrant caution when interpreting these differences.

Our study has several limitations that should be taken into account. The reliability and validity of the definition of TBI in the NEQ is unknown. While the definition implies an ICPC or ICD-10 diagnosis of head injury, the NEQ did not harvest data on specific symptoms of head injury or on diagnostic codes from the patients’ health records. Self-reported data implies risk of recall bias. It is possible that patients with disruptive symptoms and comorbid migraine look for explanations in the past and thus report a higher prevalence of premorbid TBI. The inter-rater reliability of the diagnostic evaluations was not measured. There exists no data on the overall participation rate. The generalizability of our findings to all patients in psychiatric care is thus unknown. We included patients with all BD subtypes, mood states, and pharmacological treatments. It is possible that our results would have been different if the sample had been restricted to more selected groups, e.g. patients with medication naïve first manic episodes. The Bonferroni correction for multiple testing in the bivariate analyses implies a high risk of type II errors. However, interpretation of the results should be based not only on the Bonferroni corrected p-values alone, but also on the strength of effects, prior knowledge, and biological plausibility of the associations. The design of our study makes it impossible draw conclusions about whether the findings of more disruptive symptoms and comorbid migraine are causal. Inverse causation is an alternative explanation. In regard the association between premorbid TBI and disruptive symptoms, a review found that aggression and irritability are common parts of the distal prodrome of BD (Skjelstad et al. 2010). Aggression and irritability might even characterize a prodromal subgroup of BD type II with neurocognitive deficits (Skjelstad et al. 2011), which can be hypothesized to be more prone to injuries including TBI. Residual confounding could also explain the associations. In our study, disruptive symptoms, comorbid migraine, and family history of schizophrenia were independently associated with premorbid TBI. Taken together, these variables represent a pattern of symptomatology (Hanwella and de Silva 2011), comorbidity (Fornaro et al. 2015), and heritability (Cross-Disorder Group of the Psychiatric Genomics Consortium 2013) typical for BD. We emphasize the possibility that their apparently independent associations with premorbid TBI could be a consequence of residual confounding by genetic susceptibility to BD. Examples of other factors that could have contributed to residual confounding are AD(H)D, which was probably seldom evaluated when the majority of patients in our study grew up, and psychological trauma, on which we have no data despite its known association with more severe clinical characteristics (Leverich and Post 2006) and possibly also with physical trauma including TBI.

## Conclusions

Premorbid TBI is associated with disruptive symptoms and comorbid migraine independently of the measured possible confounders including family history of mental disorders, while it is not associated with an increased hypomania/mania:depression ratio. The finding of more disruptive symptoms is in line with our hypothesis of finding similar clinical characteristics in BD with premorbid TBI as in BD due to TBI. Studies designed to draw conclusions of causal inference from observational data, e.g. by using family-based quasi-experimental designs (D’Onofrio et al. 2013), are warranted to clarify whether our findings could be explained by a continuum of pathophysiological effects of premorbid TBI across the diagnostic dichotomy of BD with premorbid TBI and BD due to TBI.

## Additional file

**Additional file 1: Table S1.** Logistic regression models on the associations between premorbid traumatic brain injury and different covariates that could attenuate its associations with the YMRS disruptive component score and comorbid migraine (values given in odds ratios with 95% confidence intervals).

## Abbreviations

BD: bipolar disorder
BRAIN: Bipolar Research And Innovation Network Norway
DSM: Diagnostic and Statistical Manual of Mental Disorders
ICD: International Classification of Diseases
ICPC: International Classification of Primary Care
IDS-C30: Inventory of Depressive Symptoms Clinician Rated 30
KMO: Kaiser-Meyer-Olkin
LOC: loss of consciousness
MINI: Mini-International Neuropsychiatric Interview
NEQ: Network Entry Questionnaire
NOS: not otherwise specified
PCA: principal component analysis
SCID I: Structured Clinical Interview for DSM IV Axis I Disorders
STEP-BD: Systematic Treatment Enhancement Program for Bipolar Disorder
TBI: traumatic brain injury
VIF: variance inflation factor
YMRS: Young Mania Rating Scale
