# Supplementary material for: Clinical characteristics of patients with bipolar disorder and premorbid traumatic brain injury: a cross-sectional study
Source: Int J Bipolar Disord. 2018 Sep 10;6:19. doi: 10.1186/s40345-018-0128-6 (PMC6162005; doi:10.1186/s40345-018-0128-6)
Supplement: Supplementary file 1 — Additional file 1: Table S1. Logistic regression models on the associations between premorbid traumatic brain injury and different covariates that could attenuate its associations with the YMRS disruptive component score and comorbid migraine (values given in odds ratios with 95% confidence intervals). [file 40345_2018_128_MOESM1_ESM.docx]

Additional Materials

Additional file 1: Table S1. Logistic regression models on the associations between premorbid traumatic brain injury and different covariates that could attenuate its associations with the YMRS disruptive component score and comorbid migraine (values given in odds ratios with 95 % confidence intervals).

|  | Model 1 (n=464) | Model 2 (n=464) |  | Model 3  (n=463) |  |  |  |  | Model 4  (n=444) |  |  |  |
| --- | --- | --- | --- | --- | --- | --- | --- | --- | --- | --- | --- | --- |
|  |  | a | b | a | b | c | d | e | a | b | c | d |
| YMRS disruptive component score | 1.6 (1.2-2.1) | 1.6 (1.2-2.2) | 1.6 (1.2-2.2) | 1.8 (1.3-2.5) | 1.6 (1.2-2.1) | 1.6 (1.2-2.1) | 1.8 (1.3-2.4) | 1.6 (1.2-2.1) | 1.6 (1.2-2.2) | 1.7 (1.3-2.2) | 1.7 (1.2-2.2) | 1.7 (1.3-2.3) |
| Comorbid migraine | 4.4 (1.8-9.9) | 4.3 (1.8-10) | 4.4 (1.9-10) | 4.0 (1.7-9.1) | 4.5 (1.9-10) | 4.4 (1.9-10) | 3.9 (1.7-9.0) | 4.4 (1.9-10) | 4.2 (1.7-10) | 4.2 (1.7-10) | 4.1 (1.6-10) | 3.9 (1.6-9.5) |

Model 1 includes age, gender, educational level, family history of depression, family history of bipolar disorder, and family history of schizophrenia as covariates (n=464). Model 2 adds and removes a) bipolar disorder type 1 and b) bipolar disorder type 2 vs. all other subtypes to the covariates in Model 1 (n=464). Model 3 adds and removes a) depression, b) euthymia, c) hypomania, d) mania, and e) mixed state vs. all other mood states to the covariates in Model 1 (n=463). Model 4 adds and removes current treatment with a) lithium, b) anticonvulsants, c) antidepressants, and d) antipsychotics vs. no current treatment with the relevant medication to the covariates in Model 1 (n=444). Variance Inflation Factor is <2 between all independent variables in all models.
